# Supplementary material for: MutationAssessor in cBioPortal
Source: bioRxiv. 2025 Aug 12:2025.08.10.669566. Preprint. [Version 1] doi: 10.1101/2025.08.10.669566 (PMC12363874; doi:10.1101/2025.08.10.669566)
Supplement: Supplement 1 [file media-1.pdf]

Supplemental Materials for

MutationAssessor in cBioPortal

Yang Su[1], Xiang Li[2], Boris Reva[3], Yevgeniy Antipin[4], Nikolaus Schultz[2], Ino de Bruijn[2], Chris Sander[1,5,6,7]

[1] Department of Systems Biology, Harvard Medical School, Boston, MA, USA

[2] Memorial Sloan Kettering Cancer Center, New York, NY, USA

[3] Departments of Obstetrics/Gynecology & Reproductive Sciences, Icahn School of Medicine at Mount Sinai, New York, NY, USA

[4] AlgoGenomics LLC, New York, NY, USA

[5] Broad Institute of Harvard and MIT, Cambridge, MA, USA

[6] CEDAR Center, Knight Cancer Center, OHSU, Portland, OR, USA

[7] Ludwig Center at Harvard; Dana-Farber/Harvard Cancer Center, Boston, MA, USA

Table S1. Predicted functional impact of single AA-substitution mutations in HUMSAVAR.

FIS Range	Predicted Functional Impact	HUMSAVAR	
		Benign	Pathogenic
FIS ≤ 2.6	Neutral	**13977**	771
2.6 < FIS ≤ 5.25	Low	15398	7608
5.25 < FIS ≤ 7	Medium	4646	18392
FIS > 7	High	315	**4345**
Total		34336	31116

Table S2. Highly recurrent somatic mutations in cancer. Somatic mutations that have been observed 300 or more times in total from all studies in cBioPortal are listed. FIS are color coded by their functional impact categories (green: neutral, yellow: low, orange: medium, red: high). GEMME scores and EVE scores for those mutations are included for comparison. Because FIS, GEMME and EVE scores are on different scales, to facilitate comparison, we show each score's percentile within the scores of all possible single AA-substitution mutations for the same protein ranked from least deleterious (0th percentile) to most deleterious (100th percentile).

Gene	UniProt Accession .Version	Protein Name	Mutant	ClinVar Clinical Label	Conservation Score	Specificity Score	FIS	%	GEMME	%	EVE	%
KRAS	P01116.1	GTPase KRas	G12D	Pathogenic	5.05	1.98	3.52	16%	-1.05	26%	0.62	59%
KRAS	P01116.1	GTPase KRas	G12V	Pathogenic	6.08	5.14	5.61	46%	-2.98	68%	0.65	64%
KRAS	P01116.1	GTPase KRas	G12C	Pathogenic	5.89	5.83	5.86	50%	-1.94	47%	0.45	38%
KRAS	P01116.1	GTPase KRas	G13D	Pathogenic	6.31	5.97	6.14	56%	-1.33	33%	0.44	37%
KRAS	P01116.1	GTPase KRas	G12A	Pathogenic	3.22	5.83	4.53	30%	-1.09	27%	0.47	40%
KRAS	P01116.1	GTPase KRas	G12R	Pathogenic	4.79	5.83	5.31	41%	-1.15	29%	0.46	39%
KRAS	P01116.1	GTPase KRas	Q61H	Pathogenic	6.43	5.93	6.18	57%	-1.79	44%	0.64	63%
KRAS	P01116.1	GTPase KRas	G12S	Pathogenic	5.49	5.14	5.32	41%	-1.73	42%	0.30	24%
KRAS	P01116.1	GTPase KRas	A146T	Pathogenic	6.95	5.96	6.46	63%	-1.57	38%	0.46	39%
KRAS	P01116.1	GTPase KRas	G13C	Pathogenic	5.96	5.97	5.96	52%	-1.78	43%	0.31	24%
KRAS	P01116.1	GTPase KRas	Q61R	Pathogenic	5.87	5.23	5.55	45%	-1.96	48%	0.60	56%
KRAS	P01116.1	GTPase KRas	Q61L	Pathogenic	5.74	3.22	4.48	29%	-1.97	48%	0.59	53%
KRAS	P01116.1	GTPase KRas	G12F		8.35	5.83	7.09	81%	-3.00	68%	0.67	68%
KRAS	P01116.1	GTPase KRas	A146V	Pathogenic	5.72	5.96	5.84	50%	-2.10	51%	0.45	38%
KRAS	P01116.1	GTPase KRas	K117N	Pathogenic	6.77	5.97	6.37	61%	-3.60	78%	0.60	55%
BRAF	P15056.4	GTPase KRas	V600E	Pathogenic	7.26	6.97	7.11	58%	-2.94	66%	0.64	73%
BRAF	P15056.4	Serine/threonine-protein kinase B-raf	G469A	Pathogenic	8.40	6.97	7.69	98%	-3.97	79%	0.71	90%
BRAF	P15056.4	Serine/threonine-protein kinase B-raf	V600K		8.35	6.97	7.66	82%	-3.26	70%	0.67	77%
BRAF	P15056.4	Serine/threonine-protein kinase B-raf	K601E	Pathogenic	6.45	6.97	6.71	49%	-2.83	65%	0.72	99%
BRAF	P15056.4	Serine/threonine-protein kinase B-raf	D594G		8.39	6.97	7.68	94%	-5.93	93%	0.72	100 %
BRAF	P15056.4	Serine/threonine-protein kinase B-raf	D594N	Pathogenic	8.39	6.97	7.68	94%	-4.08	80%	0.72	99%
PIK3CA	P42336.2	PI3-kinase subunit α	E545K	Pathogenic	7.09	5.26	6.17	72%	-2.07	59%	0.42	45%
PIK3CA	P42336.2	PI3-kinase subunit α	H1047R	Pathogenic	5.96	4.55	5.25	44%	-1.94	56%	0.36	40%

PIK3CA	P42336.2	PI3-kinase subunit α	E542K	Pathogenic	5.93	5.26	5.59	52%	-1.85	53%	0.49	51%
PIK3CA	P42336.2	PI3-kinase subunit α	R88Q	Pathogenic	5.72	5.26	5.49	49%	-2.34	64%		
PIK3CA	P42336.2	PI3-kinase subunit α	H1047L	Pathogenic	-1.16	5.24	2.04	6%	-0.96	24%	0.16	15%
PIK3CA	P42336.2	PI3-kinase subunit α	N345K	Pathogenic	6.36	5.26	5.81	57%	-1.63	48%	0.49	51%
PIK3CA	P42336.2	PI3-kinase subunit α	C420R	Pathogenic	6.38	5.26	5.82	57%	-1.34	38%	0.31	35%
PIK3CA	P42336.2	PI3-kinase subunit α	E726K	Pathogenic	3.63	5.25	4.44	27%	-0.93	22%		
PIK3CA	P42336.2	PI3-kinase subunit α	Q546K		5.27	5.26	5.26	44%	-2.29	63%	0.55	56%
PIK3CA	P42336.2	PI3-kinase subunit α	G118D	Pathogenic	6.41	5.26	5.83	58%	-1.69	49%		
PIK3CA	P42336.2	PI3-kinase subunit α	E453K	Pathogenic	7.57	5.26	6.41	79%	-1.97	56%	0.36	40%
PIK3CA	P42336.2	PI3-kinase subunit α	Q546R	Pathogenic	6.52	5.26	5.89	59%	-2.36	64%	0.77	72%
PIK3CA	P42336.2	PI3-kinase subunit α	M1043I	Pathogenic	5.25	5.25	5.25	44%	-1.71	50%	0.08	3%
PIK3CA	P42336.2	PI3-kinase subunit α	R108H		5.37	5.25	5.31	45%	-1.43	41%		
PIK3CA	P42336.2	PI3-kinase subunit α	E545G		7.09	5.26	6.17	72%	-2.40	65%	0.73	69%
PIK3CA	P42336.2	PI3-kinase subunit α	E81K	Pathogenic	6.02	5.25	5.64	53%	-2.87	72%		
PIK3CA	P42336.2	PI3-kinase subunit α	K111E	Pathogenic	5.24	5.26	5.25	44%	-1.84	53%		
PIK3CA	P42336.2	PI3-kinase subunit α	G1049R		5.34	5.25	5.29	45%	-0.93	23%	0.45	48%
TP53	P04637.4	Cellular tumor antigen p53	R175H	Pathogenic	6.99	4.43	5.71	68%	-2.84	65%	0.50	36%
TP53	P04637.4	Cellular tumor antigen p53	R248Q	Pathogenic	7.21	3.28	5.25	57%	-3.59	75%	0.43	29%
TP53	P04637.4	Cellular tumor antigen p53	R273C	Pathogenic	8.60	4.41	6.50	84%	-5.92	94%	0.85	73%
TP53	P04637.4	Cellular tumor antigen p53	R273H	Pathogenic	6.65	3.31	4.98	49%	-2.65	62%	0.39	26%
TP53	P04637.4	Cellular tumor antigen p53	R248W	Pathogenic	7.50	3.28	5.39	60%	-6.40	97%	0.94	88%
TP53	P04637.4	Cellular tumor antigen p53	R282W	Pathogenic	8.59	4.43	6.51	93%	-3.75	77%	0.92	83%
TP53	P04637.4	Cellular tumor antigen p53	Y220C	Pathogenic	8.57	4.43	6.50	83%	-3.66	75%	0.88	77%
TP53	P04637.4	Cellular tumor antigen p53	G245S	Pathogenic	7.90	4.43	6.17	76%	-3.63	75%	0.63	47%
TP53	P04637.4	Cellular tumor antigen p53	E285K	Pathogenic	6.79	4.43	5.61	66%	-4.95	87%	0.87	75%
TP53	P04637.4	Cellular tumor antigen p53	V157F	Pathogenic	4.78	4.36	4.57	42%	-2.67	63%	0.86	73%
TP53	P04637.4	Cellular tumor antigen p53	M237I	Pathogenic	8.60	4.43	6.51	96%	-2.49	60%	0.81	66%
TP53	P04637.4	Cellular tumor antigen p53	H179R	Pathogenic	7.90	3.73	5.81	69%	-6.83	99%	0.96	93%
TP53	P04637.4	Cellular tumor antigen p53	Y163C	Pathogenic	8.58	4.43	6.50	86%	-3.69	76%	0.58	43%
TP53	P04637.4	Cellular tumor antigen p53	S241F	Pathogenic	7.90	3.73	5.81	69%	-6.83	99%	0.94	89%
TP53	P04637.4	Cellular tumor antigen p53	R273L	Pathogenic	8.60	4.41	6.50	84%	-6.09	95%	0.95	91%
TP53	P04637.4	Cellular tumor antigen p53	R249S	Pathogenic	8.60	4.43	6.51	97%	-3.78	77%	0.81	67%

TP53	P04637.4	Cellular tumor antigen p53	H193R	Pathogenic	7.90	4.43	6.17	76%	-4.97	87%	0.96	93%
TP53	P04637.4	Cellular tumor antigen p53	C176F	Pathogenic	7.90	3.73	5.81	69%	-6.99	100%	0.96	94%
TP53	P04637.4	Cellular tumor antigen p53	G245D	Pathogenic	8.60	4.43	6.51	99%	-6.01	94%	0.94	88%
TP53	P04637.4	Cellular tumor antigen p53	R158L	Pathogenic	8.60	4.42	6.51	87%	-6.14	95%	0.88	77%
TP53	P04637.4	Cellular tumor antigen p53	Y234C	Pathogenic	8.52	4.43	6.48	80%	-4.17	80%	0.68	52%
TP53	P04637.4	Cellular tumor antigen p53	I195T	Pathogenic	8.57	4.43	6.50	83%	-3.97	79%	0.96	95%
TP53	P04637.4	Cellular tumor antigen p53	H179Y	Pathogenic	8.60	4.42	6.51	88%	-5.11	88%	0.94	88%
TP53	P04637.4	Cellular tumor antigen p53	R280T	Pathogenic	8.60	4.43	6.51	96%	-6.96	99%	0.99	100%
TP53	P04637.4	Cellular tumor antigen p53	C238Y	Pathogenic	8.60	4.43	6.51	98%	-6.78	98%	0.98	99%
TP53	P04637.4	Cellular tumor antigen p53	P278S	Pathogenic	8.60	4.42	6.51	89%	-3.43	73%	0.84	70%
TP53	P04637.4	Cellular tumor antigen p53	E286K	Pathogenic	7.90	4.43	6.17	76%	-6.19	96%	0.74	58%
TP53	P04637.4	Cellular tumor antigen p53	R337C	Pathogenic	4.61	4.42	4.51	41%	-1.11	36%	0.31	19%
TP53	P04637.4	Cellular tumor antigen p53	R158H	Pathogenic	6.80	3.73	5.26	57%	-2.70	63%	0.37	25%
TP53	P04637.4	Cellular tumor antigen p53	Y236C	Pathogenic	5.96	4.33	5.14	53%	-6.06	95%	0.63	47%
TP53	P04637.4	Cellular tumor antigen p53	V173L	Pathogenic	8.60	4.43	6.51	99%	-2.21	55%	0.67	51%
TP53	P04637.4	Cellular tumor antigen p53	V272M	Pathogenic	6.90	4.43	5.67	67%	-3.75	77%	0.96	94%
TP53	P04637.4	Cellular tumor antigen p53	S127F	Pathogenic	8.60	4.43	6.51	100%	-6.80	98%	0.87	76%
TP53	P04637.4	Cellular tumor antigen p53	K132N	Pathogenic	7.90	4.43	6.17	75%	-4.43	83%	0.84	71%
TP53	P04637.4	Cellular tumor antigen p53	C176Y	Pathogenic	8.60	4.42	6.51	88%	-6.99	100%	0.96	95%
TP53	P04637.4	Cellular tumor antigen p53	G266R	Pathogenic	8.60	4.43	6.51	96%	-6.58	98%	0.95	91%
TP53	P04637.4	Cellular tumor antigen p53	P152L	Pathogenic	7.90	4.43	6.17	75%	-2.21	55%	0.74	58%
TP53	P04637.4	Cellular tumor antigen p53	V173M	Pathogenic	8.60	4.43	6.51	99%	-4.31	82%	0.96	93%
TP53	P04637.4	Cellular tumor antigen p53	H214R	Pathogenic	6.32	4.43	5.37	59%	-3.58	74%	0.77	63%
TP53	P04637.4	Cellular tumor antigen p53	V216M	Pathogenic	8.58	4.43	6.51	87%	-3.54	74%	0.89	78%
TP53	P04637.4	Cellular tumor antigen p53	G266E	Pathogenic	8.60	4.43	6.51	96%	-5.15	89%	0.91	81%
TP53	P04637.4	Cellular tumor antigen p53	L194R	Pathogenic	8.60	4.43	6.51	94%	-6.65	98%	0.97	97%
TP53	P04637.4	Cellular tumor antigen p53	C135Y	Pathogenic	8.57	4.43	6.50	85%	-6.17	95%	0.97	97%
TP53	P04637.4	Cellular tumor antigen p53	G245C	Pathogenic	8.60	4.43	6.51	99%	-5.64	92%	0.95	92%
TP53	P04637.4	Cellular tumor antigen p53	R280K	Pathogenic	8.60	4.43	6.51	96%	-2.68	63%	0.96	94%
TP53	P04637.4	Cellular tumor antigen p53	P151S	Pathogenic	6.79	4.43	5.61	66%	-2.19	55%	0.77	62%
TP53	P04637.4	Cellular tumor antigen p53	G245V	Pathogenic	8.60	4.43	6.51	99%	-6.11	95%	0.96	94%

TP53	P04637.4	Cellular tumor antigen p53	K132R	Pathogenic	8.60	4.43	6.51	97%	-2.46	59%	0.54	39%
TP53	P04637.4	Cellular tumor antigen p53	C135F	Pathogenic	8.57	4.43	6.50	85%	-6.17	95%	0.98	98%
TP53	P04637.4	Cellular tumor antigen p53	C141Y	Pathogenic	7.18	3.73	5.45	61%	-4.26	81%	0.96	94%
TP53	P04637.4	Cellular tumor antigen p53	G154V	Pathogenic	8.57	4.43	6.50	84%	-5.18	89%	0.85	73%
TP53	P04637.4	Cellular tumor antigen p53	R248L	Pathogenic	8.60	4.38	6.49	81%	-5.94	94%	0.86	74%
TP53	P04637.4	Cellular tumor antigen p53	C275Y	Pathogenic	8.60	4.42	6.51	89%	-6.98	99%	0.98	100 %
TP53	P04637.4	Cellular tumor antigen p53	Y205C	Pathogenic	8.59	4.43	6.51	91%	-6.41	97%	0.89	79%
TP53	P04637.4	Cellular tumor antigen p53	V272L	Pathogenic	6.90	4.43	5.67	67%	-2.02	52%	0.70	54%
TP53	P04637.4	Cellular tumor antigen p53	C277F		8.60	4.43	6.51	98%	-4.59	84%	0.93	87%
TP53	P04637.4	Cellular tumor antigen p53	C242F	Pathogenic	8.60	4.43	6.51	99%	-6.85	99%	0.98	100 %
TP53	P04637.4	Cellular tumor antigen p53	A159V	Pathogenic	6.80	4.43	5.62	67%	-2.46	59%	0.40	28%
TP53	P04637.4	Cellular tumor antigen p53	P278L	Pathogenic	7.90	3.73	5.82	69%	-5.45	90%	0.77	62%
TP53	P04637.4	Cellular tumor antigen p53	R110L	Pathogenic	3.63	4.41	4.02	32%	-1.19	38%	0.62	46%
TP53	P04637.4	Cellular tumor antigen p53	C238F	Pathogenic	8.60	4.43	6.51	98%	-6.78	98%	0.97	97%
TP53	P04637.4	Cellular tumor antigen p53	G266V	Pathogenic	8.60	4.43	6.51	96%	-6.84	99%	0.97	96%
TP53	P04637.4	Cellular tumor antigen p53	G244D	Pathogenic	8.60	4.43	6.51	98%	-3.56	74%	0.70	54%
TP53	P04637.4	Cellular tumor antigen p53	E271K	Pathogenic	8.59	4.43	6.51	92%	-3.45	73%	0.79	64%
TP53	P04637.4	Cellular tumor antigen p53	R213Q	Pathogenic	7.50	3.73	5.61	66%	-3.32	72%	0.81	67%
TP53	P04637.4	Cellular tumor antigen p53	E258K	Pathogenic	8.60	4.43	6.51	97%	-5.53	91%	0.95	92%
TP53	P04637.4	Cellular tumor antigen p53	C275F	Pathogenic	8.60	4.42	6.51	89%	-6.98	99%	0.98	100 %
TP53	P04637.4	Cellular tumor antigen p53	H193Y	Pathogenic	8.60	4.43	6.51	99%	-4.10	80%	0.89	79%
TP53	P04637.4	Cellular tumor antigen p53	N239D	Pathogenic	7.88	4.43	6.16	74%	-3.34	72%	0.93	87%
TP53	P04637.4	Cellular tumor antigen p53	A161T	Pathogenic	6.77	4.43	5.60	66%	-2.65	62%	0.89	78%
IDH1	O75874.2	Isocitrate dehydrogenase [NADP] cytoplasmic	R132H	Pathogenic	7.16	4.65	5.90	36%	-2.14	53%	0.75	57%
IDH1	O75874.2	Isocitrate dehydrogenase [NADP] cytoplasmic	R132C	Pathogenic	8.41	5.34	6.88	53%	-2.36	57%	0.93	97%
IDH1	O75874.2	Isocitrate dehydrogenase [NADP] cytoplasmic	R132G		9.10	6.04	7.57	73%	-4.63	89%	0.92	92%
IDH1	O75874.2	Isocitrate dehydrogenase [NADP] cytoplasmic	R132L		9.80	6.04	7.92	93%	-3.97	81%	0.93	97%
EGFR	P00533.2	Epidermal growth factor receptor	L858R	Pathogenic	8.57	5.43	7.00	81%	-5.60	92%	0.85	88%
EGFR	P00533.2	Epidermal growth factor receptor	T790M	Pathogenic	8.80	5.43	7.12	96%	-2.97	72%	0.83	81%
EGFR	P00533.2	Epidermal growth factor receptor	A289V	Pathogenic	8.54	5.43	6.98	79%	-3.86	81%	0.70	63%
EGFR	P00533.2	Epidermal growth factor receptor	G719A	Pathogenic	8.11	5.43	6.77	75%	-3.97	82%	0.86	91%

EGFR	P00533.2	Epidermal growth factor receptor	L861Q	Pathogenic	8.81	5.43	7.12	99%	-2.05	58%	0.85	87%
EGFR	P00533.2	Epidermal growth factor receptor	S768I	Pathogenic	7.46	5.43	6.44	65%	-3.18	74%	0.77	72%
JAK2	O60674.2	Tyrosine-protein kinase JAK2	V617F	Pathogenic	6.64	4.73	5.69	49%	-3.38	61%	0.74	65%
NRAS	P01111.1	GTPase NRas	Q61R	Pathogenic	5.91	4.84	5.38	46%	-1.70	49%	0.60	48%
NRAS	P01111.1	GTPase NRas	Q61K	Pathogenic	6.81	4.84	5.83	54%	-1.91	54%	0.66	57%
NRAS	P01111.1	GTPase NRas	G12D	Pathogenic	5.05	4.83	4.94	38%	-1.11	35%	0.70	64%
NRAS	P01111.1	GTPase NRas	Q61L	Pathogenic	5.77	5.54	5.65	50%	-1.41	42%	0.57	45%
NRAS	P01111.1	GTPase NRas	G13D	Pathogenic	6.31	5.54	5.92	56%	-1.13	35%	0.61	49%
NRAS	P01111.1	GTPase NRas	Q61H		6.50	4.84	5.67	51%	-1.51	45%	0.64	54%
NRAS	P01111.1	GTPase NRas	G13R		7.09	5.54	6.31	64%	-2.67	68%	0.53	41%
SF3B1	O75533.3	Splicing factor 3B subunit 1	K700E	Pathogenic	7.29	6.18	6.74	51%	-3.09	70%		
SF3B1	O75533.3	Splicing factor 3B subunit 1	K666N		7.91	6.18	7.05	67%	-4.10	84%		
SF3B1	O75533.3	Splicing factor 3B subunit 1	R625C	Pathogenic	8.00	6.18	7.09	99%	-4.09	84%		
U2AF1	Q01081.3	Splicing factor U2AF 35 kDa subunit	S34F	Pathogenic	6.78	4.70	5.74	45%	-2.65	64%	0.90	83%
U2AF1	Q01081.3	Splicing factor U2AF 35 kDa subunit	Q157P		5.88	5.81	5.84	47%	-3.01	69%	0.45	45%
DNMT3A	Q9Y6K1.4	DNA (cytosine-5)-methyltransferase 3A	R882H	Pathogenic	8.27	5.75	7.01	75%	-0.57	17%	0.62	75%
DNMT3A	Q9Y6K1.4	DNA (cytosine-5)-methyltransferase 3A	R882C	Pathogenic	8.27	5.75	7.01	75%	-2.07	54%	0.64	82%
GNAS	P63092.1	Guanine nucleotide-binding protein G(s) subunit α isoforms short	R201H	Pathogenic	7.67	6.32	7.00	75%			0.48	41%
GNAS	P63092.1	Guanine nucleotide-binding protein G(s) subunit α isoforms short	R201C		7.57	5.63	6.60	66%			0.63	56%
IDH2	P48735.2	Isocitrate dehydrogenase [NADP], mitochondrial	R140Q	Pathogenic	6.89	4.81	5.85	33%	-2.78	58%	0.89	65%
IDH2	P48735.2	Isocitrate dehydrogenase [NADP], mitochondrial	R172K		9.09	5.51	7.30	60%	-2.81	58%	0.97	97%
SRSF2	Q01130.4	Serine/arginine-rich splicing factor 2	P95H		4.83	4.64	4.74	44%	-3.72	82%		
SRSF2	Q01130.4	Serine/arginine-rich splicing factor 2	P95L		4.51	4.64	4.58	39%	-2.61	66%		
SRSF2	Q01130.4	Serine/arginine-rich splicing factor 2	P95R		2.72	4.64	3.68	17%	-2.67	68%		
ERBB2	P04626.1	Receptor tyrosine-protein kinase erbB-2	S310F	Pathogenic	5.14	5.07	5.10	35%	-2.87	68%		
ERBB2	P04626.1	Receptor tyrosine-protein kinase erbB-2	R678Q		2.23	5.75	3.99	21%	-0.99	29%		
ERBB2	P04626.1	Receptor tyrosine-protein kinase erbB-2	L755S		8.59	5.76	7.17	81%	-4.80	87%		
ERBB2	P04626.1	Receptor tyrosine-protein kinase erbB-2	V842I		5.20	5.76	5.48	41%	-1.86	50%		
ERBB2	P04626.1	Receptor tyrosine-protein kinase erbB-2	V777L		0.78	5.76	3.27	14%	-1.53	42%		
ERBB2	P04626.1	Receptor tyrosine-protein kinase erbB-2	S310Y	Pathogenic	6.78	5.76	6.27	57%	-2.29	59%		
SMAD4	Q13485.1	Mothers against decapentaplegic homolog 4	R361H	Pathogenic	7.79	5.48	6.63	99%	-2.22	53%	0.71	86%

SMAD4	Q13485.1	Mothers against decapentaplegic homolog 4	R361C	Pathogenic	7.79	5.48	6.63	99%	-3.82	75%	0.73	99%
FBXW7	Q969H0.1	F-box/WD repeat-containing protein 7	R465C	Pathogenic	8.17	6.83	7.50	99%	-2.59	48%		
FBXW7	Q969H0.1	F-box/WD repeat-containing protein 7	R465H		8.17	6.83	7.50	99%	-3.86	72%		
FBXW7	Q969H0.1	F-box/WD repeat-containing protein 7	R505C		8.16	6.83	7.50	95%	-3.27	61%		
FBXW7	Q969H0.1	F-box/WD repeat-containing protein 7	R479Q		8.16	6.83	7.50	94%	-3.51	66%		
FBXW7	Q969H0.1	F-box/WD repeat-containing protein 7	R505G		7.47	6.83	7.15	64%	-4.42	80%		
CTNNB1	P35222.1	Catenin β-1	S37F		8.09	5.92	7.01	72%	-3.74	78%	0.82	83%
CTNNB1	P35222.1	Catenin β-1	T41A		7.37	5.92	6.65	54%	-2.16	53%	0.78	73%
CTNNB1	P35222.1	Catenin β-1	S45F		6.07	5.92	6.00	35%	-3.60	76%	0.78	73%
CTNNB1	P35222.1	Catenin β-1	S37C		8.09	5.92	7.01	72%	-2.32	56%	0.81	80%
CTNNB1	P35222.1	Catenin β-1	S45P		7.86	5.92	6.89	65%	-1.18	33%	0.73	63%
CTNNB1	P35222.1	Catenin β-1	S33C		7.40	5.92	6.66	54%	-2.39	57%	0.77	72%
CTNNB1	P35222.1	Catenin β-1	S33F		8.09	5.92	7.01	72%	-3.87	80%	0.84	90%
CTNNB1	P35222.1	Catenin β-1	D32Y		8.08	5.92	7.00	71%	-3.84	80%	0.78	73%
CTNNB1	P35222.1	Catenin β-1	T41I		8.06	5.92	6.99	70%	-3.59	76%	0.78	73%
CTNNB1	P35222.1	Catenin β-1	G34R		8.09	5.92	7.01	72%	-4.39	88%	0.82	84%
BCOR	Q6W2J9.1	BCL-6 corepressor	N1459S		5.36	5.07	5.21	36%	-2.91	53%	0.59	76%
PTEN	P60484.1	Phosphatidylinositol 3,4,5-trisphosphate 3-phosphatase and dual-specificity protein phosphatase PTEN	R130Q		6.33	4.19	5.26	47%	-4.44	90%	0.73	99%
PTEN	P60484.1	Phosphatidylinositol 3,4,5-trisphosphate 3-phosphatase and dual-specificity protein phosphatase PTEN	R130G		7.71	5.29	6.50	93%	-7.67	100 %	0.72	97%
PTEN	P60484.1	Phosphatidylinositol 3,4,5-trisphosphate 3-phosphatase and dual-specificity protein phosphatase PTEN	R173C		7.72	5.30	6.51	99%	-4.97	94%	0.70	90%
ESR1	P03372.2	Estrogen receptor	D538G		5.46	4.84	5.15	41%	-4.26	81%	0.55	55%
ESR1	P03372.2	Estrogen receptor	Y537S		4.49	4.84	4.67	34%	-2.41	49%	0.50	51%
CDKN2A	P42771.2	Cyclin-dependent kinase inhibitor 2A	H83Y		7.58	4.43	6.01	98%	-3.99	82%		
PPP2R1A	P30153.4	Serine/threonine-protein phosphatase 2A 65 kDa regulatory subunit A α isoform	P179R		7.73	4.84	6.28	66%	-5.60	96%	0.99	89%
PPP2R1A	P30153.4	Serine/threonine-protein phosphatase 2A 65 kDa regulatory subunit A α isoform	R183W	Pathogenic	7.71	5.53	6.62	76%	-4.59	90%	0.99	90%
PPP2R1A	P30153.4	Serine/threonine-protein phosphatase 2A 65 kDa regulatory subunit A α isoform	S256F		7.72	5.53	6.63	77%	-2.14	62%	0.99	87%
ERBB3	P21860.1	Receptor tyrosine-protein kinase erbB-3	V104M		4.02	5.61	4.82	32%	-0.63	15%	0.35	46%
FGFR2	P21802.1	Fibroblast growth factor receptor 2	S252W	Pathogenic	9.38	5.25	7.32	84%	-2.89	77%	0.64	67%

FGFR2	P21802.1	Fibroblast growth factor receptor 2	N549K		7.84	5.25	6.54	63%	-2.83	77%	0.70	72%
HRAS	P01112.1	GTPase HRas	Q61R	Pathogenic	6.03	5.84	5.94	53%	-2.14	52%	0.61	50%
SPOP	O43791.1	Speckle-type POZ protein	F133L		3.90	5.05	4.48	19%	-1.04	20%	0.49	53%
SPOP	O43791.1	Speckle-type POZ protein	F133V		6.08	5.05	5.56	53%	-2.31	56%	0.57	69%
GNA11	P29992.2	Guanine nucleotide-binding protein subunit α-11	Q209L	Pathogenic	6.80	5.14	5.97	66%	-3.47	81%	0.84	74%
H3-3A	P84243.2	Histone H3.3	K28M		5.20	6.12	5.66	43%	-0.84	23%	0.56	33%
RAC1	P63000.1	Ras-related C3 botulinum toxin substrate 1	P29S		4.55	5.12	4.84	43%	-1.31	48%	0.56	32%
XPO1	O14980.1	Exportin-1	E571K		6.83	6.41	6.62	59%	-3.50	71%		
ATM	Q13315.4	Serine-protein kinase ATM	R337C		6.98	4.30	5.64	80%	-5.71	95%	0.84	93%
KIT	P10721.1	Mast/stem cell growth factor receptor Kit	V559G		7.73	5.46	6.60	74%	-3.16	77%	0.79	75%

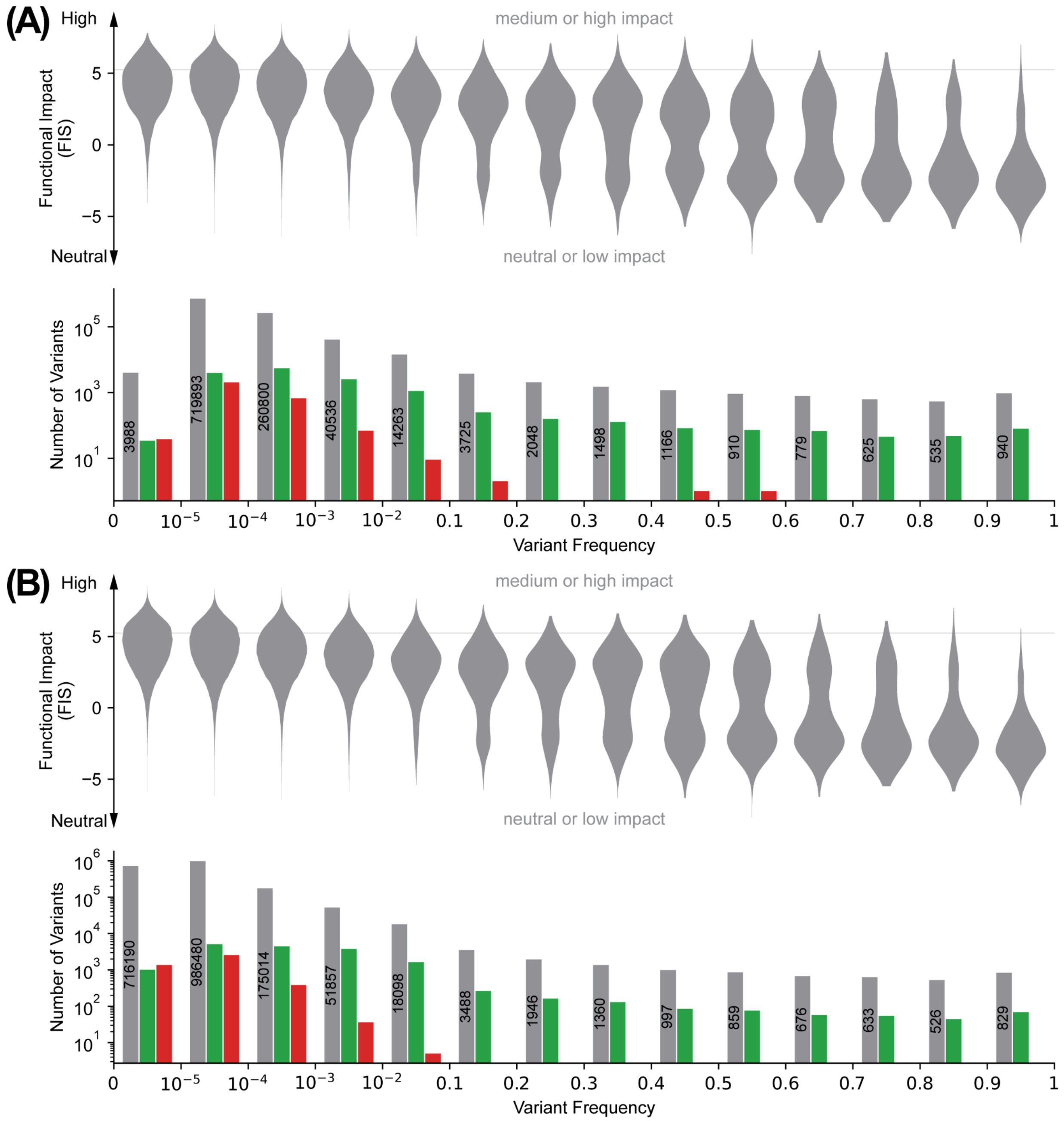

Figure S1. Frequencies and distributions of predicted functional impact of variants in the population. Functional impact (FIS) and variant frequencies of AA-changing single substitution variants in the NCBI ALFA dataset (A) and gnomAD whole genome sequencing dataset (B). Variants are binned by their frequencies calculated using the respective dataset. For each bin, the number of variants therein (lower, gray), the number of variants that are annotated as benign or pathogenic in ClinVar (lower, green or red), and their FIS distribution (upper) are shown. Horizontal lines indicate the boundary between neutral/low impact and medium/high impact (FIS = 5.25). Vertical lines indicate where the horizontal axis scale changes.

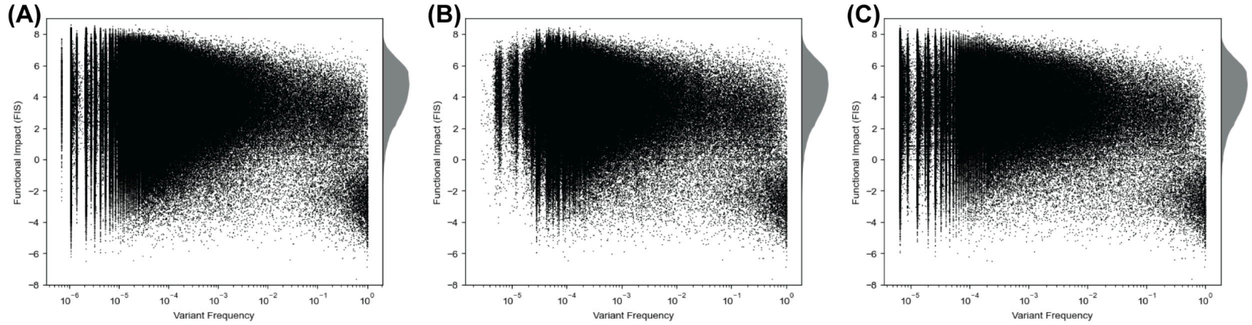

Figure S2. FIS and variant frequency in the human population. Each dot represents a unique AA-changing single substitution variant in the UK Biobank 500k whole genome sequencing dataset (A), NCBI ALFA (B), and gnomAD whole genome sequencing dataset (C). A density plot of FIS is shown on the margin of each scatter plot.

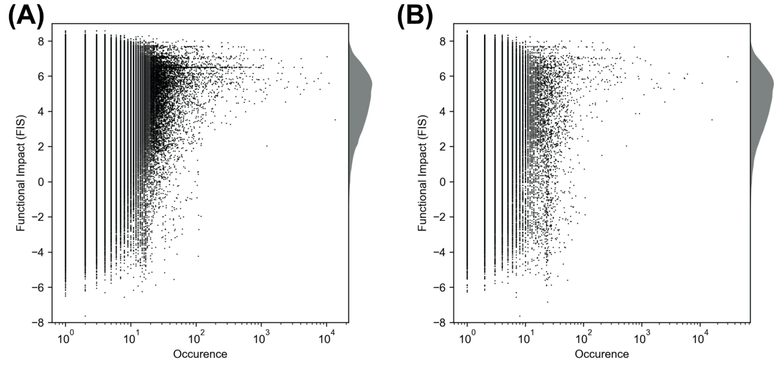

Figure S3. FIS and the number of occurrences of the somatic variants in cBioPortal (A) and COSMIC (B). Each dot represents a unique mutation. A density plot of FIS is shown on the margin.

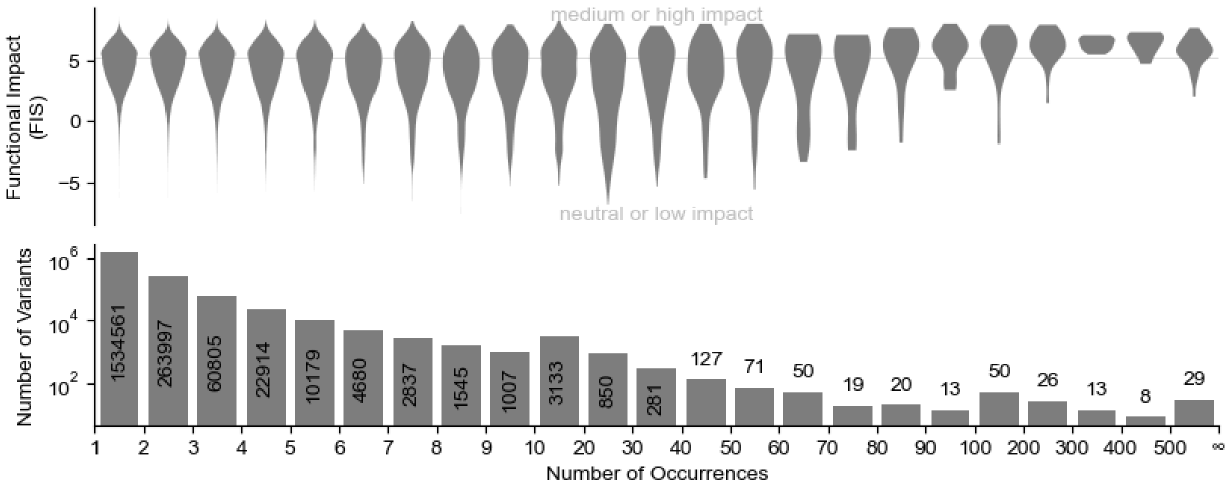

Figure S4. FIS distribution of cancer mutations from COSMIC Cancer Mutation Census. Distributions of FIS (**A**) and the number of unique mutations (**B**) stratified by occurrence.
